# Neuropsychiatric symptoms in at-risk groups for AD dementia and their association with worry and AD biomarkers—results from the DELCODE study

**DOI:** 10.1186/s13195-020-00701-7

**Published:** 2020-10-16

**Authors:** Lena Sannemann, Ann-Katrin Schild, Slawek Altenstein, Claudia Bartels, Frederic Brosseron, Katharina Buerger, Nicoleta Carmen Cosma, Klaus Fliessbach, Silka Dawn Freiesleben, Wenzel Glanz, Michael T. Heneka, Daniel Janowitz, Ingo Kilimann, Xenia Kobeleva, Christoph Laske, Coraline D. Metzger, Matthias H. J. Munk, Robert Perneczky, Oliver Peters, Alexandra Polcher, Josef Priller, Boris Rauchmann, Christina Rösch, Janna Rudolph, Anja Schneider, Annika Spottke, Eike Jakob Spruth, Stefan Teipel, Ruth Vukovich, Michael Wagner, Jens Wiltfang, Steffen Wolfsgruber, Emrah Duezel, Frank Jessen, for the DELCODE Study Group

**Affiliations:** 1 Department of Psychiatry, Medical Faculty, University of Cologne, Kerpener Strasse 62, 50924 Cologne, Germany; 2 German Center for Neurodegenerative Diseases (DZNE), Berlin, Germany; 3 Department of Psychiatry and Psychotherapy, Charité, Charitéplatz 1, 10117 Berlin, Germany; 4 German Center for Neurodegenerative Diseases (DZNE), Goettingen, Germany; 5 Department of Psychiatry and Psychotherapy, University Medical Center Goettingen, University of Goettingen, Von-Siebold-Str. 5, 37075 Goettingen, Germany; 6 German Center for Neurodegenerative Diseases (DZNE), Bonn, Venusberg-Campus 1, 53127 Bonn, Germany; 7 Department for Neurodegenerative Diseases and Geriatric Psychiatry, University Hospital Bonn, Venusberg-Campus 1, 53127 Bonn, Germany; 8 German Center for Neurodegenerative Diseases (DZNE, Munich), Feodor-Lynen-Strasse 17, 81377 Munich, Germany; 9 Institute for Stroke and Dementia Research (ISD), University Hospital, LMU Munich, Feodor-Lynen-Strasse 17, 81377 Munich, Germany; 10 Charité – Universitätsmedizin Berlin, corporate member of Freie Universität Berlin, Humboldt-Universität zu Berlin, and Berlin Institute of Health, Institute of Psychiatry and Psychotherapy, Hindenburgdamm 30, 12203 Berlin, Germany; 11 German Center for Neurodegenerative Diseases (DZNE), Magdeburg, Germany; 12 German Center for Neurodegenerative Diseases (DZNE), Rostock, Germany; 13 Department of Psychosomatic Medicine, Rostock University Medical Center, Gehlsheimer Str. 20, 18147 Rostock, Germany; 14 Department of Neurology, University of Bonn, Venusberg-Campus 1, 53127 Bonn, Germany; 15 German Center for Neurodegenerative Diseases (DZNE), Tübingen, Germany; 16 Section for Dementia Research, Hertie Institute for Clinical Brain Research and Department of Psychiatry and Psychotherapy, University of Tübingen, Tübingen, Germany; 17 Institute of Cognitive Neurology and Dementia Research (IKND), Otto-von-Guericke University, Magdeburg, Germany; 18 Department of Psychiatry and Psychotherapy, Otto-von-Guericke University, Magdeburg, Germany; 19 Department of Psychiatry and Psychotherapy, University Hospital, LMU Munich, Munich, Germany; 20 Munich Cluster for Systems Neurology (SyNergy) Munich, Munich, Germany; 21 Ageing Epidemiology Research Unit (AGE), School of Public Health, Imperial College London, London, UK; 22 Neurosciences and Signaling Group, Institute of Biomedicine (iBiMED), Department of Medical Sciences, University of Aveiro, Aveiro, Portugal; 23 Excellence Cluster on Cellular Stress Responses in Aging-Associated Diseases (CECAD), University of Cologne, Joseph-Stelzmann-Strasse 26, 50931 Köln, Germany

**Keywords:** Alzheimer’s disease, Neuropsychiatric symptoms, Subjective cognitive decline, Amyloid

## Abstract

**Background:**

Early identification of individuals at risk of dementia is mandatory to implement prevention strategies and design clinical trials that target early disease stages. Subjective cognitive decline (SCD) and neuropsychiatric symptoms (NPS) have been proposed as potential markers for early manifestation of Alzheimer’s disease (AD). We aimed to investigate the frequency of NPS in SCD, in other at-risk groups, in healthy controls (CO), and in AD patients, and to test the association of NPS with AD biomarkers, with a particular focus on cognitively unimpaired participants with or without SCD-related worries.

**Methods:**

We analyzed data of *n* = 687 participants from the German DZNE Longitudinal Cognitive Impairment and Dementia (DELCODE) study, including the diagnostic groups SCD (*n* = 242), mild cognitive impairment (MCI, *n* = 115), AD (*n* = 77), CO (*n* = 209), and first-degree relatives of AD patients (REL, *n* = 44). The Neuropsychiatric Inventory Questionnaire (NPI-Q), Geriatric Depression Scale (GDS-15), and Geriatric Anxiety Inventory (GAI-SF) were used to assess NPS. We examined differences of NPS frequency between diagnostic groups. Logistic regression analyses were carried out to further investigate the relationship between NPS and cerebrospinal fluid (CSF) AD biomarkers, focusing on a subsample of cognitively unimpaired participants (SCD, REL, and CO), who were further differentiated based on reported worries.

**Results:**

The numbers of reported NPS, depression scores, and anxiety scores were significantly higher in subjects with SCD compared to CO. The quantity of reported NPS in subjects with SCD was lower compared to the MCI and AD group. In cognitively unimpaired subjects with worries, low Aß42 was associated with higher rates of reporting two or more NPS (OR 0.998, 95% CI 0.996–1.000, *p* < .05).

**Conclusion:**

These findings give insight into the prevalence of NPS in different diagnostic groups, including SCD and healthy controls. NPS based on informant report seem to be associated with underlying AD pathology in cognitively unimpaired participants who worry about cognitive decline.

**Trial registration:**

German Clinical Trials Register DRKS00007966. Registered 4 May 2015.

## Introduction

In light of the growing significance of preventive interventions and clinical trials that target individuals at the very early stages of Alzheimer’s disease (AD), early detection of the disease has become a main research area. We know today that pathological changes in AD begin years before the onset of cognitive decline [1] and that identifying individuals in prodromal or at-risk states of AD is of major importance in order to use this timeframe for prevention of cognitive decline. While cognitive symptoms are traditionally regarded as the core feature of AD, neuropsychiatric symptoms (NPS) can be observed frequently along the AD continuum, including prodromal stages of the disease [2]. Among the most common NPS in patients with mild cognitive impairment (MCI) and early AD are depression, agitation, and apathy [2], but also anxiety and irritability that show a high prevalence especially in MCI [3, 4]. In cognitively normal persons of the National Alzheimer’s Coordinating Center (NACC) database who progressed to dementia over the median follow-up of 4.7 years, the most prevalent NPS were depression, irritability, sleep disturbances, appetite changes, and anxiety [5].

NPS have been reported to appear as the first symptoms of impending dementia in many patients [6, 7]. Recent evidence supports the idea that subtle NPS in pre-clinical stages may act as potential heralds of progression to cognitive decline [8–11] and could therefore be used to improve early detection of the disease. A study by Masters, Morris, and Roe on cognitively normal participants found that NPS appear earlier in subjects who later progress to MCI or dementia as indicated by a Clinical Dementia Rating (CDR) > 0 compared to those who remain at CDR 0 [12].

The assumption that pathological changes result in behavioral symptoms and that NPS can be regarded as early manifestations of neurodegenerative diseases resulted in the introduction of mild behavioral impairment (MBI) as a diagnostic construct to identify at-risk patients for dementia [13]. While the possibility of concurrent cognitive impairment is included in the research criteria, the authors clearly differentiate between MCI and MBI.

There have been ambiguous reports about the predictive ability of NPS. In a longitudinal cohort study on community dwelling subjects, overall NPS were not associated with cognitive decline over 2 years in cognitively healthy and MCI subjects [14]. However, results from a Mexican cohort study showed that certain NPS were independent predictors of incident dementia after 3 years [15] and data from the Mayo Clinic Study of Aging indicated that agitation, apathy, anxiety, irritability, and depression in cognitively normal persons predicted incident MCI [10]. Results from a recent longitudinal study on cognitively normal persons showed that psychotic, affective, and agitation symptoms predicted Alzheimer’s dementia, among other dementia subtypes [5].

While the presence of NPS in MCI and AD patients and their impact on patients and caregivers are well documented, less is known about the occurrence in people with subjective cognitive decline (SCD). SCD is defined as self-experienced memory decline in the absence of objective cognitive impairment on neuropsychological tests [16]. There is accumulating evidence that SCD is associated with an increased risk for progression to MCI and AD dementia [17–21], which might be up to twice as high compared to people without subjective complaints [22]. Results from the AgeCoDe study showed that in subjects with longitudinally stable SCD, the risk of incident dementia was doubled compared to healthy controls without SCD [23]. From a biological perspective, AD-related pathological changes in the CSF, namely decreased concentration of Aß42 and increased concentration of tau, are more common in individuals with SCD compared to healthy controls without memory concerns, and predict clinical progression [17, 24–26].

However, not everyone who experiences SCD progresses to clinical stages of the disease. Certain features have been reported as potentially enriching factors for preclinical AD. These features are referred to as “SCD plus” criteria and include, among others, SCD that is accompanied by worry or concern about the experienced cognitive decline [16, 27]. Worry about memory decline has been shown to increase the risk of conversion to MCI or AD [16, 19, 28, 29] and might reflect underlying AD pathology.

The relevance of these noncognitive symptoms, especially in prodromal stages of the disease, is also mirrored by the new research framework of the National Institute on Aging and Alzheimer’s Association (NIA-AA) work group [30]. The framework introduces “stage 2,” which is defined as a stage of transitional cognitive decline in individuals of the Alzheimer’s continuum. It acknowledges that early signs of AD might not necessarily present as cognitive deficits. NPS may coexist or even represent the main change. Hence, a cognitively asymptomatic individual with positive biomarker evidence of AD can be classified as stage 2 based on the occurrence of NPS alone.

In the present study, we aim to explore the presence of NPS along the continuum of AD in a large German multicenter cohort. In addition, we test the association of subtle NPS and AD biomarkers in cognitively healthy individuals with and without worry about self-perceived memory decline.

## Method

### Participants

For this cross-sectional analysis, baseline data of *N* = 687 participants from the ongoing DZNE Longitudinal Cognitive Impairment and Dementia (DELCODE) study were included. DELCODE is an observational longitudinal multicenter study in Germany that enrolls subjects with SCD, MCI patients, AD dementia patients, healthy control subjects (CO), and first-degree relatives of patients with a documented diagnosis of AD dementia (REL). Participants were 60 years or older, German-speaking, and had no current major depressive episode and no current or past major psychiatric disorders. All participants were accompanied by study partners that acted as informants. Study partners agreed to provide information about the participant over the course of the study. Most frequently, spouses (53.1%), children (20.4%), or friends (11.6%) acted as informants.

Initial clinical assessment was performed at the participating memory clinic that defined the DELCODE entry diagnosis for the patient groups (SCD, MCI, AD). The SCD group was defined by the presence of self-experienced cognitive decline in the absence of objective cognitive impairment (− 1.5 SD) on all subtests of the CERAD neuropsychological battery [25]. Healthy controls and first-degree relatives of AD patients were recruited via harmonized newspaper advertisements. Controls were screened with regard to SCD criteria and only included if they did not express concern about their memory. Detailed inclusion and exclusion criteria as well as the definition of patient groups have been described before [25].

The study was approved by the institutional review boards (IRB) and ethical committees of each of the participating DELCODE sites. All participants provided written informed consent prior to inclusion.

### Clinical measures

All participants underwent clinical examination as well as neuropsychological testing and risk factor assessment. The Mini-Mental State Examination (MMSE) score and Clinical Dementia Rating (CDR) were obtained from each participant. For description of the full protocol, please refer to Jessen et al. [21]. The factor structure of 27 variables from the neuropsychological tests battery was tested with confirmatory factor analysis (CFA) and yielded five factors (memory [MEM], executive function [EXEC], visuospatial abilities [VIS], working memory [WM] and language [LANG] [31];).

In addition to the observation of cognitive status, the DELCODE design included the assessment of neuropsychiatric symptoms using the Neuropsychiatric Inventory Questionnaire (NPI-Q), the 15-item short form of Geriatric Depression Scale (GDS-15), and the short form of the Geriatric Anxiety Inventory (GAI-SF). These questionnaires are well-established and frequently used in research studies and clinical trials to provide a brief assessment of neuropsychiatric symptomatology, as well as depressive and anxiety symptoms in older patients. While information on the GAI-SF was available for *n* = 686, NPI-Q and the GDS-15 were completed for *n* = 665, respectively.

The NPI-Q is an informant-based rating scale that evaluates 12 neuropsychiatric symptoms [32], namely delusions, hallucinations, agitation, depression, anxiety, euphoria, apathy, disinhibition, irritability, aberrant motor behavior, sleep disturbance, and changes in appetite. For each item, the informant indicates whether the symptom has been present during the last month (“yes” or “no”) and rates its severity on a 3-point Likert scale for this time period (mild, moderate, severe). Higher total scores (0–36) indicate more neuropsychiatric symptoms. For this study, we focused on the number of NPS that were reported during the last month.

Subthreshold symptoms of depression and anxiety were quantified using the GDS-15 and GAI-SF. The 15-item GDS screens for depression in older adults and covers the period of the preceding week [33, 34]. The GAI-SF is a 5-item screening measure for anxiety symptoms in older people [35]. Both scales require “yes” or “no” answers for each item, with higher scores indicating the presence of more depressive symptoms (GDS, 0–15) or more anxiety symptoms (GAIS-SF, 0–5). Akin to previous studies, we decided to consider a total score of ≥ 1 as the cutoff for indication of subsyndromal symptoms [36].

Worry about cognitive decline was recorded with the question “Are you worried that you might have a memory or thinking problem?”, which was assessed before administering the Everyday Cognition questionnaire (ECog [37]).

### CSF AD biomarker assessment

In a subset of *n* = 317, AD biomarker information obtained at the baseline investigation was available. Commercially available kits according to vendor specifications were used: V-PLEX Aβ Peptide Panel 1 (6E10) Kit (K15200E) and V-PLEX Human Total Tau Kit (K151LAE) (Mesoscale Diagnostics LLC, Rockville, USA), and Innotest Phospho-Tau(181P) (81,581; Fujirebio Germany GmbH, Hannover, Germany). Samples from all DELCODE sites were analyzed centrally in the laboratory of the DZNE in Bonn.

The cutoffs for normal and abnormal concentrations of Aß42 (< 496 pg/ml) and for the ratio Aß42/Aß40 (< 0.09) were derived from the literature, which applied to the respective assays [25, 38]. The cutoff values for tau (> 470 pg/ml) and p-tau (> 57 pg/ml) were established locally (Bonn) based on clinical non-impaired control samples.

### Statistical analysis

All statistical analyses were performed with IBM SPSS Statistics 25.0 (IBM Corp., Armonk, NY) for Windows.

We tested for differences between diagnostic groups at baseline with regard to demographic variables and clinical and neuropsychological measures, as well as biomarker data using ANOVAs and chi-square tests. In addition, Bonferroni-corrected post hoc tests were applied to identify significant differences of each group compared to the control group.

The GDS-15 total score, GAI-SF total score, and the number of reported NPS on the NPI-Q were compared between diagnostic groups by using a Kruskal-Wallis nonparametric test. Pairwise comparisons were applied via Dunn-Bonferroni post hoc tests.

The relationship between NPS and AD biomarkers was analyzed in subjects with available CSF biomarkers (*n* = 317). As expected due to the exclusion of participants with major depressive or psychiatric disorders, the data on neuropsychiatric symptoms showed a positive skew. Therefore, binary logistic regression analyses were carried out using the number of confirmed NPI-Q items as the dichotomized outcome variables. For the first logistic regression, the outcome was defined as one or more reported NPS (no NPS, 0; one or more NPS, 1). For the second analysis, a more conservative threshold of two or more NPS was applied (less than two NPS, 0; two or more NPS, 1). CSF biomarkers were added as non-dichotomized, continuous predictors and age, sex, education, and the memory factor score (MEM) as covariates to control for demographic differences and cognitive decline. All explanatory variables were included in the model simultaneously. The analyses were repeated for the GDS and GAI-SF, applying the same cutoffs of the respective total score. These cutoffs were chosen to reflect mild changes in behavior, as suggested by the NIA-AA research framework [30], that do not reach the threshold for clinical relevance.

Additionally, we performed a single item analysis based on the NPI-Q items that were reported most frequently in our sample. Binary logistic regression analyses were carried out as described above with absence (0) or presence (1) of agitation, depression, anxiety, apathy, irritability, and sleep disturbances as the dependent variables.

To examine whether subtle NPS might be a correlate of early pathological changes of AD before the onset of cognitive decline, the analyses were repeated in a subsample of cognitively healthy participants (CO, subjects with SCD and first-degree relatives of AD patients). This “cognitively healthy” group was then split into “worriers” and “non-worriers” based on the ECog to capture the SCD-plus criterion of worry or concern about the self-experienced decline. As worriers have been reported to have an increased risk of conversion to MCI and AD, our aim was to examine the relationship between NPS and AD biomarkers in cognitively healthy subjects with or without worry about their memory.

Since our aim was to analyze the association of AD biomarkers and NPS in an exploratory and not hypothesis-driven way, we report unadjusted *p* values for the logistic regression analyses.

## Results

The demographic data as well as clinical and cognitive characteristics of the sample are presented in Table 1. The characteristics of the sample are similar to the first baseline data of 394 DELCODE participants that have been described elsewhere [25].

**Table 1:** Description of the DELCODE sample at baseline

	Total (*n* = 687)	CO (*n* = 209)	SCD (*n* = 242)	MCI (*n* = 115)	AD (*n* = 77)	REL (*n* = 44)	*F* value/*χ*^2^ value
Sex (female), *n* (*%*)	351 (51.1)	120 (57.4)	117 (48.3)	42 (36.5)**	43 (55.8)	29 (65.9)	18.4, *p* = .001
Age, *mean* (*SD*)	70.4 (5.9)	68.8 (5.3)	71.1 (5.7)***	72.1 (5.3)***	73.7 (6.7)***	64.6 (3.9)***	27.32 *p* < .001
Education years, *mean* (*SD*)	14.4 (3.0)	14.8 (2.8)	14.8 (3.1)	14.0 (3.0)	13.2 (3.3)**	14.1 (2.6)	5.72, *p* < .001
MMSE score, *mean* (*SD*)	28.4 (2.4)	29.4 (0.9)	29.2 (1.1)	27.8 (1.8)***	23.5 (3.1)***	29.3 (1.1)	251.02, *p* < .001
Expresses worry about memory (ECog), *n* (*%*)	386 (58.7)	27 (13.2)	193 (83.2)***	93 (85.3)***	56 (80.0)***	17 (39.5)***	282.73, *p* < .001
MEM, *mean* (*SD*)	4.02 × 10^−7^ (0.98)	0.60 (0.39)	0.34 (0.47)***	−0.68 (0.67)***	−1.99 (0.62)***	0.51 (0.61)	446.38, *p* < .001
CSF biomarkers	***n* = 317**	***n* = 76**	***n* = 104**	***n* = 74**	***n* = 39**	***n* = 24**	
Aß42 (pg/ml), *mean* (*SD*)	677.7 (317.3)	851.8 (301.6)	715.5 (310.6)*	568.4 (278.8)***	390.5 (137.2)***	767 (289.9)	20.98, *p* < .001
Aß42 < 496 pg/ml (*n*, %)	110 (34.7)	10 (13.2)	25 (24.0)	39 (52.7)***	31 (79.5)***	5 (20.8)	67.93, *p* < .001
t-tau (pg/ml), mean (SD)	489.8 (265.5)	389.9 (160.1)	408.4 (192.2)	556.2 (258.7)***	844.8 (338.8)***	372.8 (103.9)	35.85, *p* < .001
t-tau > 470 pg/ml, *n* (%)	127 (40.2)	18 (24.0)	31 (29.9)	40 (54.1)***	34 (87.2)***	4 (16.7)	60.11, *p* < .001
p-tau 181 (pg/ml), mean (SD)	60.4 (30.3)	51.3 (18.4)	51.73 (23.9)	67.4 (31.8)**	96.1 (38.6)***	46.8 (12.3)	24.87, *p* < .001
p-tau 181 > 57 pg/ml, *n* (%)	135 (44.4)	24 (31.6)	30 (30.6)	43 (61.4)**	33 (86.8)***	5 (22.7)	52.74, *p* < .001

Bonferroni-adjusted post hoc *p* values in comparison to the control group: *p < .0125, **p < .003, ***p < .0003

*Aβ42* beta-amyloid 42, *AD* Alzheimer’s disease, *CSF* cerebrospinal fluid, *ECog* Everyday Cognition scale, *CO* healthy controls, *MCI* mild cognitive impairment, *MEM* memory factor score, *MMSE* Mini-Mental-State Examination, *p-tau* phospho-tau, *REL* first-degree relatives of AD patients, *SCD* subjective cognitive decline, *SD* standard deviation, *t-tau* total tau

**Fig. 1:**
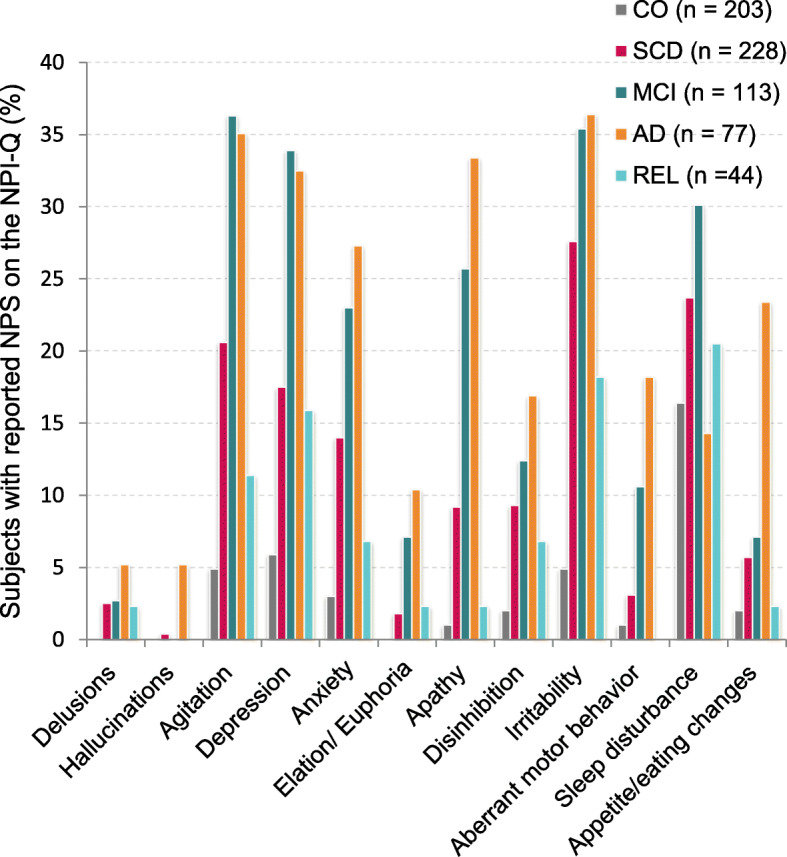
Prevalence of NPS per group as measured by the NPI-Q in the DZNE DELCODE study. The graph depicts the percentage of participants with reported symptoms in each NPI-Q domain. In subjects with SCD, irritability, sleep disturbances, and agitation were the most frequently reported NPS, followed by anxiety and depression

The prevalence of neuropsychiatric symptoms as measured by the NPI-Q is shown in Fig. 1. In subjects with SCD, 53.5% study informants reported the presence of at least one neuropsychiatric symptom. Only a quarter of the healthy controls showed one or more NPS, whereas the majority of study informants reported at least one NPS in MCI and AD patients. The most frequently reported NPS in SCD subjects were irritability (27.6%), sleep disturbance (23.7%), and agitation (20.6%). The presence of depression and anxiety was reported by 17.5% and 14.0%, respectively.

Table 2 presents the description of neuropsychiatric symptoms in the DELCODE sample. As expected due to the exclusion of participants with major depressive episodes or major psychiatric disorders, GDS-15 and GAI-SF scores were below clinical thresholds in most cases. In subjects with SCD, 76.0% indicated subsyndromal depressive symptoms on the GDS-15 and 65.1% exhibited subsyndromal anxiety symptoms.

**Table 2:** Neuropsychiatric symptoms in the whole DELCODE sample and per diagnostic group at baseline

	Total		CO		SCD		MCI		AD		REL	
	*n*	*M*, *SD*	*n*	*M*, *SD*	*n*	*M*, *SD*	*n*	*M*, *SD*	*n*	*M*, *SD*	*n*	*M*, *SD*
Number of reported NPI-Q Items	665	1.3 (1.8)	203	0.4 (0.8)	228	1.4 (1.8)	113	2.2 (2.0)	77	2.6 (2.5)	44	0.9 (1.3)
GDS-15 total score	664	1.5 (1.8)	204	0.7 (1.3)	233	1.8 (1.8)	109	2.1 (1.9)	75	2.2 (1.8)	43	1.0 (1.5)
GAI-SF total score	686	1.0 (1.1)	209	0.7 (0.8)	241	1.2 (1.2)	115	1.0 (1.1)	77	1.1 (1.2)	44	1.2 (1.1)
	*n*	%	*n*	%	*n*	%	*n*	%	*n*	%	*n*	%
NPS ≥ 1	334	50.2	53	26.1	122	53.5	83	73.5	56	72.7	20	45.5
NPS ≥ 2	218	32.8	23	11.3	70	30.7	68	60.2	48	62.3	9	20.5
GDS-15 score ≥ 1	427	64.3	74	36.3	177	76.0	91	83.5	62	82.7	23	53.5
GAI-SF score ≥ 1	401	58.5	103	49.3	157	65.1	65	56.5	46	59.7	30	68.2

*AD* Alzheimer’s disease, *GDS-15* Geriatric Depression Scale 15-item version, *GAI-SF* Geriatric Anxiety Inventory—Short Form, *CO* healthy controls, *MCI* mild cognitive impairment, *NPI-Q* Neuropsychiatric Inventory Questionnaire, *NPS* neuropsychiatric symptoms, *REL* first-degree relatives of AD patients, *SCD* subjective cognitive decline

**Fig. 2:**
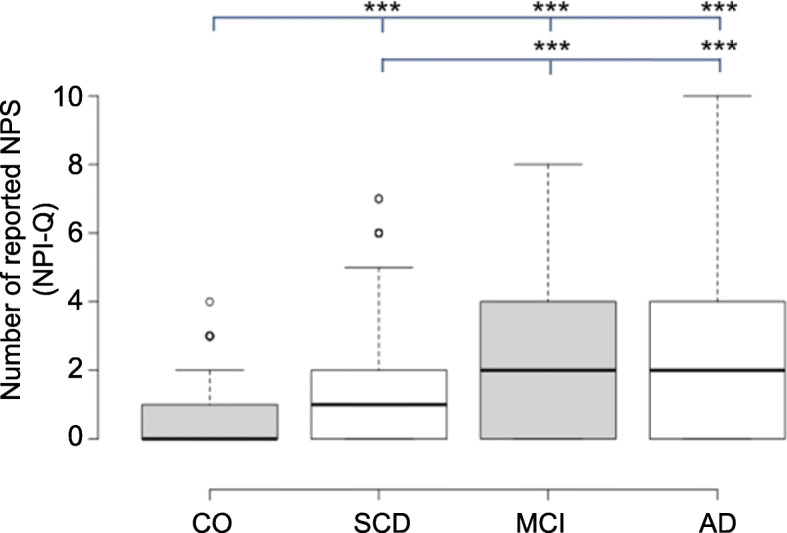
Boxplot of the number of reported NPS for each group, showing the median. *p < .05; **p < .01; ***p < .001. Whiskers extend 1.5 times the interquartile range from the 25th and 75th percentiles; outliers are represented by dots. The number of reported NPS in SCD subjects is higher compared to CO but significantly lower than in MCI and AD patients. CO reported significantly less NPS compared to SCD, MCI, and AD patients

The Kolmogorov-Smirnov test revealed that the total scores for GDS-15, GAI-SF and the sum of reported NPI-Q symptoms were non-normally distributed. Groups differed significantly in the number of reported NPS domains (*H* = 120.70, *p* < .001), in the GDS-15 total score (*H* = 126.26, *p* < .001), and the GAI-SF total score (*H* = 24.89, *p* < .001). Dunn-Bonferroni post hoc tests revealed an increased quantity of reported NPS in the SCD participants compared to the CO group (*p* < .001), while they reported less than both the MCI (*p* < .001) and AD group (*p* < .001). First-degree relatives of AD patients reported less NPS than the patient groups (MCI *p* < .001, AD *p* < .001). The results are plotted in Fig. 2.

For GDS-15, post hoc analysis revealed that healthy controls scored significantly lower than SCD subjects (*p* < .001), MCI patients (*p* < .001), and AD patients (*p* < .001). Akin to the NPI-Q domains, relatives of AD patients scored lower on the GDS-15 than MCI (*p* = .001) and AD patients (*p* < .001), but also compared to subjects with SCD (*p* < .01). There was no significant difference between SCD subjects and patient groups. Anxiety symptoms as measured by the GAI-SF were lower in CO compared to SCD subjects (*p* < .001), yet there was no statistically significant difference between other groups.

The results of the logistic regression analyses on the association with AD biomarkers are presented in Tables 3 and 4. They show that for the whole sample, a reduction of Aß42 and a lower score on the memory factor were associated with reporting one or more NPS on the NPI-Q. We found no significant predictor for the presence of at least one NPS in the subgroup of cognitively healthy individuals. To examine whether the presence of worry about one’s cognitive abilities as a potentially enriching factor for preclinical AD might influence outcomes, the sample of cognitively healthy participants was divided into “worriers” and “non-worriers.” However, no significant predictor was found in either group.

**Table 3:** Regression coefficients and odds ratios (95% confidence intervals) from binary logistic regression to predict the presence of ≥ 1 NPS on the NPI-Q

	Whole sample (*n* = 296)	Cognitively healthy (CO + SCD + REL) (*n* = 190)	Cognitively healthy worrier (*n* = 100)	Cognitively healthy non-worrier (*n* = 88)
Predictors	OR (95% CI), ***p*** value	OR (95% CI), ***p*** value	OR (95% CI), ***p*** value	OR (95% CI), ***p*** value
Age	1.002 (0.958–1.048), 0.921	1.028 (0.971–1.089), 0.343	1.019 (0.943–1.102), 0.631	1.015 (0.914–1.127), 0.787
Sex	0.972 (0.583–1.621), 0.913	1.108 (0.560–2.191), 0.769	1.099 (0.434–2.782), 0.843	1.235 (0.397–3.847), 0.716
Education	0.968 (0.888–1.056), 0.467	1.018 (0.911–1.137), 0.757	1.070 (0.924–1.238), 0.365	0.904 (0.729–1.121), 0.357
MEM	0.597 (0.414–0.862), 0.006**	0.486 (0.226–1.048), 0.066	0.662 (0.258–1.698), 0.391	0.293 (0.069–1.244), 0.096
Aß42 (pg/ml)	0.999 (0.998–1.000), 0.030*	1.000 (0.998–1.001), 0.353	0.999 (0.998–1.000), 0.124	1.001 (0.999–1.003), 0.180
t-tau (pg/ml)	0.999 (0.996–1.001), 0.243	0.999 (0.995–1.003), 0.571	0.999 (0.994–1.004), 0.780	1.000 (0.993–1.000), 0.960
p-tau (pg/ml)	1.013 (0.993–1.034), 0.211	1.011 (0.981–1.042), 0.482	1.017 (0.977–1.058), 0.409	0.987 (0.928–1.050), 0.688

Note: * p < .05, ** p < .01. Aβ42 beta-amyloid 42, *CO* healthy controls, *CI* confidence interval, *MEM* memory factor score, *OR* odds ratio, *p-tau* phospho-tau, *REL* first-degree relatives of AD patients, *SCD* subjective cognitive decline, *t-tau* total tau

**Table 4:** Regression coefficients and odds ratios (95% confidence intervals) from binary logistic regression to predict the presence of ≥ 2 NPS on the NPI-Q

	Whole sample (*n* = 296)	Cognitively healthy (CO + SCD + REL) (*n* = 190)	Cognitively healthy worrier (*n* = 100)	Cognitively healthy non-worrier (*n* = 88)
Predictors	OR (95% CI), ***p*** value	OR (95% CI), ***p*** value	OR (95% CI), ***p*** value	OR (95% CI), ***p*** value
Age	1.023 (0.976–1.073), 0.343	1.059 (0.989–1.135), 0.101	1.078 (0.989–1.175), 0.088	1.004 (0.877–1.151), 0.950
Sex	0.818 (0.476–1.407), 0.469	0.808 (0.358–1.826), 0.609	0.910 (0.333–2.483), 0.854	0.604 (0.144–2.538), 0.491
Education	0.989 (0.902–1.084), 0.809	1.044 (0.917–1.189), 0.517	1.029 (0.885–1.197), 0.709	1.011 (0.774–1.320), 0.938
MEM	0.686 (0.478–0.985), 0.041*	0.686 (0.300–1.571), 0.373	1.014 (0.396–2.596), 0.978	0.396 (0.068–2.299), 0.302
Aß42 (pg/ml)	0.998 (0.997–0.999), 0.002**	0.999 (0.998–1.000), 0.108	0.998 (0.996–1.000), 0.040*	1.001 (0.998–1.004), 0.402
t-tau (pg/ml)	0.998 (0.995–1.000), 0.073	0.997 (0.993–1.002), 0.238	1.000 (0.997–1.004), 0.881	0.992 (0.983–1.002), 0.135
p-tau (pg/ml)	1.023 (1.002–1.045), 0.033*	1.023 (0.986–1.061), 0.230	1.001 (0.978–1.025), 0.934	1.030 (0.945–1.122), 0.502

Note: * p < .05, ** p < .01. Aβ42 beta-amyloid 42, *CO* healthy controls, *CI* confidence interval, *MEM* memory factor score, *OR* odds ratio, *p-tau* phospho-tau, *REL* first-degree relatives of AD patients, *SCD* subjective cognitive decline, *t-tau* total tau

Similarly, reporting at least two NPS appeared to be more common in participants with lower Aß42 and MEM score, but also in those with increased p-tau. While there were no significant predictors in the cognitively healthy subgroup, decreased Aß42 was significantly associated with a higher risk of two or more confirmed NPS in worriers based on the ECog classification, but not in non-worriers.

The analyses of the GDS and GAI-SF did not replicate these result patterns. Most notably, an increase of p-tau was associated with GDS scores ≥ 1 and ≥ 2, but only in the whole sample. Detailed results are presented in the Additional file 1, Table S1 and S2.

Single-item analysis of the most frequently reported NPI-Q domains allowed us to examine whether certain NPS might have a stronger association with AD biomarkers compared to the total number of reported NPS. In summary, a reduction of Aß42 was associated with a higher risk of agitation (OR 0.998, 95% CI 0.997–1.000, *p* < .01), depression (OR 0.998, 95% CI 0.997–0.999, *p* < .01), and anxiety (OR 0.999, 95% CI 0.997–1.000, *p* < .05), but not with apathy, irritability, and sleep disturbances in the whole sample. In the subsample of cognitively healthy participants, this relationship was found for the depression (OR 0.998, 95% CI 0.996–1.000, *p* < .05) and anxiety (OR 0.997, 95% CI 0.995–1.000, *p* < .05) domains. We again differentiated between cognitively healthy worriers and non-worriers. Lower levels of Aß42 significantly increased the risk of agitation (OR 0.997, 95% CI 0.995–1.000, *p* < .05), anxiety (OR 0.996, 95% CI 0.992–0.999, *p* < .05), and irritability (OR 0.998, 95% CI 0.996–1.000, *p* < .05) only in cognitively healthy worriers. Detailed results can be found in the Additional file 1, Table S3.

## Discussion

We examined the prevalence of NPS in a large German observational cohort study in individuals at risk of AD and patients with MCI and AD dementia and the association of NPS with AD biomarkers. We found that NPS were more prevalent in subjects with SCD compared to healthy controls, but less prevalent compared to MCI and AD patients. Pathological changes of Aß42 seemed to be associated with the informant report of ≥ 1 or ≥ 2 NPS on the NPI-Q. In cognitively healthy subjects who worry about their memory, decreased Aß42 increased the likelihood of two or more reported NPS. For the GDS and GAI-SF, these results were not replicated.

The occurrence of NPS in at-risk subjects is comparable to findings by Masters et al. [12], who observed three phases of NPS over time. Irritability, depression, and sleep disturbances appeared first, followed by anxiety, appetite changes, agitation, and apathy. Subsequently, elation/euphoria, motor disturbances, hallucinations, delusions, and disinhibition formed the third phase. While the authors noted that the order of appearance was similar for patients who developed a CDR rating > 0 at follow-up and those who remained stable, NPS appeared earlier in participants who later progressed. Our results show a similar trend with irritability, sleep disturbances, and depression being among the most frequently reported NPS in SCD subjects. Depression and anxiety symptoms, albeit subsyndromal, were significantly pronounced in individuals with SCD compared to healthy controls. It is however important to note that question 10 of the GDS-15 asks about memory deficits. Out of all SCD subjects that scored ≥ 1 on the GDS-15, 55.9% confirmed this question and thus were of the opinion that they had more difficulties with their memory compared to others.

Our results give further insight into the relationship between AD biomarkers and the presence of NPS. Combining all patient groups, participants with decreased CSF Aß42 levels were more likely to exhibit one or more NPS on the NPI-Q. This was not the case in the subgroup of cognitively healthy individuals. Although this subgroup likely includes subjects with preclinical AD, others may never develop AD and this heterogeneity might have masked significant effects of underlying AD pathology. In fact, previous research has shown that SCD may be caused by a number of underlying medical conditions, for example, metabolic diseases, endocrine diseases, psychiatric conditions, and sleep disorders, but also by certain personality traits such as neuroticism [39, 40]. While subtle NPS may exist or even cause SCD in these cases, progression to MCI or dementia is less likely and we would not expect to see an association with AD pathology.

In contrast, when we looked at cognitively healthy worriers, who are at higher risk for preclinical AD [16], we found that Aß42 independently predicted the presence of two or more NPS on the NPI-Q. However, the effects we observed were small and need to be replicated in a larger sample to substantiate the relationship. It is also important to note that these results were based on caregiver-report, which may be biased by incomplete or inaccurate perceptions of NPS, especially in mild AD stages [41].

For single domains of the NPI-Q, ambiguous results were observed. In cognitively healthy worriers, lower Aß42 was a significant predictor for the presence of anxiety, agitation, and irritability. The presence of depressive symptoms was predicted by lowered Aß42 in the whole sample and the subgroup of cognitively healthy, but not after splitting the sample into worriers and non-worriers. Again, while this might indicate an association between pathological changes in the CSF and increased expression of certain NPS, we only observed very small OR, limiting the informative value of our findings. The results add to findings from the Massachusetts Alzheimer’s Disease Research Center longitudinal cohort. In a Cox analysis, the authors identified greater symptoms of depression, irritability, and agitation as predictors for more rapid disease progression in a group of healthy controls, subjects with subjective cognitive decline and MCI patients [20]. Likewise, depression and anxiety have been shown to be more commonly reported in cognitively healthy subjects who later progressed to MCI or AD over a period of 4 years [11]. Taking into account the results of our analyses, changes of Aß42 might manifest as early symptoms on these neuropsychiatric domains in particular. However, longitudinal analyses are necessary to explore the causality of this relationship, especially considering the concept of NPS as early manifestations of frontotemporal dementia (FTD) [13, 42–44].

Recent findings from an interim analysis of the DELCODE study (*n* = 205) demonstrated that the number of fulfilled SCD-plus criteria was significantly associated with measures of amyloid pathology [27]. With regard to single SCD-plus features, experienced decline in memory, onset of SCD within the last 5 years, and confirmation by an informant were associated with amyloid pathology, whereas the relationship with worry almost reached significance level. For this study, we did not consider any other SCD-plus criteria, but for future analyses, it will be interesting to explore the relationship between SCD-plus features, NPS, and AD biomarkers.

In addition, in the aforementioned study, it was reported that 10% of the SCD subjects did not express worries, even though concerns about self-experienced cognitive decline reported in the initial memory clinic assessment formed an inclusion criterion for the study. In contrast, a proportion of participants in the control group expressed worrisome decline at baseline [27]. Our analysis on a larger dataset confirmed this finding on the ECog—specifically, 16.8% of subjects with SCD did not report any worries, whereas 13.2% of CO and 39.5% of first-degree relatives reported worries. It is therefore important to consider the stability of worries over time. Consequently, the consistency of SCD has recently been proposed as a new addition to the SCD-plus criteria [40, 45]. In a study by van Harten et al. [29], who likewise used the ECog to differentiate between participants who reported worry and those who did not, self-reported worry was associated with a 1.87-fold higher risk of MCI in cognitively unimpaired subjects. Their results indicate that consistency of SCD and worry are independent predictors of clinical progression to MCI. Consistently reported worrisome SCD has also been shown to be related to increased risk of incident AD dementia [23].

## Limitations

There are limitations to our study. Whereas for the whole sample, a reduction of CSF Aß42 levels increased the risk of reporting at least one NPS, we did not observe a statistically significant relationship between AD biomarkers and the presence of at least one NPS in the subgroup of cognitively healthy worriers. A possible explanation is that the NPI-Q rates the presence of NPS over the last month, which is a relatively short timeframe that can easily be influenced by variability of exterior conditions or medical situation. It is therefore plausible that a criterion of one or more NPS might be of limited sensitivity to detect AD-related behavioral changes. In contrast, the MBI criteria use a definition of behavioral changes that persist for at least 6 months and are severe enough to have at least minimal negative impact on interpersonal relationships, other aspects of social functioning, or ability to perform in the workplace [13].

Additionally, the NPI-Q is an informant-based questionnaire and subtle NPS might easily be missed, especially if the study partner does not live with the participant. A study by Banks et al. [11] demonstrated that a study partner’s overall report about behavioral symptoms did not predict decline to MCI or dementia in healthy older adults, whereas self-reported symptoms did. Partner-reported symptoms increased over time in those who progressed, indicating that informant-based rating of NPS might be more accurate in later stages of the disease [11]. To further investigate the role of subtle NPS, an instrument that does not only rely on the study partner’s report might provide more valid information in cognitively healthy participants. For example, the recently developed “Mild Behavioral Impairment Checklist” (MBI-C) can be completed by participants, study partners or clinicians [46].

While the GDS and GAI-SF are self-reported measures, they might be prone to social desirability bias, which could influence the results. Especially in later stages of AD, patients may lack awareness of behavioral changes or comprehension of the questions itself might be limited. In addition, the GAI-SF only consists of 5 questions. While the short form has been developed to detect Generalized Anxiety Disorder (GAD), it is not clear whether subtle anxiety symptoms are adequately assessed with such a limited number of questions [35].

Another limitation is related to the analysis method. Even though continuous data was available for the regression analyses, we decided to dichotomize the NPI-Q scores and perform logistic regression analyses because of the severe skew in the data. This resulted in a loss of information, which limits the generalizability of our results.

## Conclusions

In the present study, we analyzed preliminary baseline data based on 687 participants in the DELCODE study. We found that NPS were more prevalent in subjects with SCD compared to healthy controls, but less prevalent compared to MCI and AD patients. In addition, depression and anxiety scores were higher in subjects with SCD compared to healthy controls.

Our results give insight into the association between AD biomarkers and NPS and provide further evidence for worry as an enriching factor for preclinical AD. Longitudinal analyses will be necessary to explore how the presence of worry and NPS in at-risk groups for AD dementia may accelerate clinical progression and how these features can be used to improve early detection of AD and prediction of disease progression.

## Supplementary information

**Additional file 1: Table S1.** Results from binary logistic regression to predict a GDS total score ≥ 1 (A) and ≥ 2 (B). **Table S2.** Results from binary logistic regression to predict a GAI-SF total score ≥ 1 (A) and ≥ 2 (B). **Table S3.** Results from binary logistic regression to predict the presence of agitation, depression, anxiety, apathy, irritability and sleep disturbances on the NPI-Q.

## Data Availability

The data that support this study are not publically available, but may be provided upon reasonable request.

## Abbreviations

AD: Alzheimer’s disease
AgeCoDe: German Study on Ageing, Cognition and Dementia in Primary Care Patients
CDR: Clinical Dementia Rating
CERAD: Consortium to Establish a Registry for Alzheimer’s Disease
CFA: Confirmatory factor analysis
CO: Healthy controls
DELCODE: DZNE-Longitudinal Cognitive Impairment and Dementia Study
CSF: Cerebrospinal fluid
DZNE: German Centre for Neurodegenerative Diseases (Deutsches Zentrum für Neurodegenerative Erkrankungen)
ECog: Everyday Cognition questionnaire
FTD: Frontotemporal dementia
GAI-SF: Geriatric Anxiety Inventory—short form
GDS: Geriatric Depression Scale
MBI: Mild behavioral impairment
MCI: Mild cognitive impairment
MMSE: Mini-Mental State Examination
NACC: National Alzheimer’s Coordinating Center
NIA-AA: National Institute on Aging and Alzheimer’s Association
NPI-Q: Neuropsychiatric Inventory Questionnaire
NPS: Neuropsychiatric symptoms
REL: First-degree relatives of patients with Alzheimer’s disease
SCD: Subjective cognitive decline
SPSS: Statistical Package for the Social Sciences—25th edition
