# Supplementary material for: Neuropsychiatric symptoms in at-risk groups for AD dementia and their association with worry and AD biomarkers—results from the DELCODE study
Source: Alzheimers Res Ther. 2020 Oct 16;12:131. doi: 10.1186/s13195-020-00701-7 (PMC7566134; doi:10.1186/s13195-020-00701-7)
Supplement: Supplementary file 1 — Additional file 1: Table S1. Results from binary logistic regression to predict a GDS total score ≥ 1 (A) and ≥ 2 (B). Table S2. Results from binary logistic regression to predict a GAI-SF total score ≥ 1 (A) and ≥ 2 (B). Table S3. Results from binary logistic regression to predict the presence of agitation, depression, anxiety, apathy, irritability and sleep disturbances on the NPI-Q. [file 13195_2020_701_MOESM1_ESM.pdf]

Table S1. A) Regression Coefficients and Odds Ratios (95% Confidence Intervals) from binary logistic regression to predict a GDS score ≥ 1

Predictors	Whole sample (n = 296)	Cognitively healthy (CO+SCD+REL) (n = 190)	Cognitively healthy worrier (n = 100)	Cognitively healthy non-worrier (n = 88)
	**OR (95% CI), p-value**	**OR (95% CI), p-value**	**OR (95% CI), p-value**	**OR (95% CI), p-value**
Age	1.041 (0.991 – 1.092), 0.107	1.039 (0.978 – 1.103), 0.214	1.013 (0.927 – 1.108), 0.772	1.072 (0.976 – 1.178), 0.146
Sex	0.801 (0.465 – 1.377), 0.422	0.944 (0.476 – 1.876), 0.870	0.870 (0.295 – 2.563), 0.800	0.978 (0.361 – 2.650), 0.965
Education	0.987 (0.899 - 1.083), 0.775	0.973 (0.868 – 1.091), 0.639	0.949 (0.804 – 1.121), 0.542	0.994 (0.825 – 1.197), 0.946
MEM	0.718 (0.483 – 0.067), 0.101	0.565 (0.250 – 1.279), 0.171	0.521 (0.149 – 1.825), 0.308	0.579 (0.167 – 2.007), 0.386
Aß42 (pg/ml)	0.999 (0.998 – 1.000), 0.173	0.999 (0.998 – 1.000), 0.049*	0.999 (0.997 – 1.000), 0.152	0.999 (0.998 – 1.001), 0.414
t-tau (pg/ml)	0.997 (0.995– 1.000), 0.045*	0.996 (0.993 – 1.000), 0.064	0.995 (0.990 – 1.001), 0.083	0.997 (0.991 – 1.003), 0.379
p-tau (pg/ml)	1.023 (1.000 – 1.046), 0.047*	1.030 (0.977 – 1.064), 0.077	1.042 (0.992 – 1.094), 0.099	1.020 (0.968 – 1.075), 0.448

Note. * *p* < .05. *Aβ42* beta-amyloid 42; *CO* healthy controls; *CI* Confidence Interval; *MEM* Memory factor score; *OR* Odds ratio; *p-tau* phospho-tau; *REL* first degree relatives of AD patients; *SCD* subjective cognitive decline; *t-tau* total tau

Table S1. B) Regression Coefficients and Odds Ratios (95% Confidence Intervals) from binary logistic regression to predict a GDS score ≥ 2

Predictors	Whole sample (n = 296)	Cognitively healthy (CO+SCD+REL) (n = 190)	Cognitively healthy worrier (n = 100)	Cognitively healthy non-worrier (n = 88)
	**OR (95% CI), p-value**	**OR (95% CI), p-value**	**OR (95% CI), p-value**	**OR (95% CI), p-value**
Age	1.022 (0.977 – 1.069), 0.351	1.033 (0.971 – 1.099), 0.303	1.056 (0.973 – 1.146), 0.193	1.033 (0.905 – 1.179), 0.632
Sex	0.795 (0.476 – 1.328), 0.380	0.600 (0.294 – 1.228), 0.162	0.489 (0.190 – 1.263), 0.140	0.611 (0.142 – 2.630), 0.508
Education	1.053 (0.965 – 1.150), 0.242	1.065 (0.947 – 1.199), 0.293	0.989 (0.853 – 1.146), 0.883	1.205 (0.922 – 1.576), 0.172
MEM	0.733 (0.517 – 1.041), 0.083	0.659 (0.308 – 1.412), 0.284	0.902 (0.3622 – 2.252), 0.826	0.233 (0.039 – 2.388), 0.110
Aß42 (pg/ml)	0.999 (0.998 – 1.000), 0.169	0.999 (0.998 – 1.000), 0.077	0.999 (0.997– 1.000), 0.083	1.000 (0.998 – 1.003), 0.670
t-tau (pg/ml)	0.998 (0.995 – 1.000), 0.062	0.996 (0.992 – 1.000), 0.050	0.995(0.989 – 1.000), 0.039*	0.996 (0.988 – 1.005), 0.400
p-tau (pg/ml)	1.022 (1.001 – 1.043), 0.036*	1.031 (0.998 – 1.066), 0.068	1.041 (0.999 – 1.084), 0.054	1.025 (0.951 – 1.104), 0.518

Note. * *p* < .05. *Aβ42* beta-amyloid 42; *CO* healthy controls; *CI* Confidence Interval; *MEM* Memory factor score; *OR* Odds ratio; *p-tau* phospho-tau; *REL* first degree relatives of AD patients; *SCD* subjective cognitive decline; *t-tau* total tau

Table S2. A) Regression Coefficients and Odds Ratios (95% Confidence Intervals) from binary logistic regression to predict a GAI-SF score ≥ 1

	Whole sample (n = 302)	Cognitively healthy (CO+SCD+REL) (n = 194)	Cognitively healthy worrier (n = 104)	Cognitively healthy non-worrier (n = 88)
Predictors	**OR (95% CI), p-value**	**OR (95% CI), p-value**	**OR (95% CI), p-value**	**OR (95% CI), p-value**
Age	0.988 (0.947 – 1.032), 0.593	1.000 (0.946 – 1.057), 0.992	0.962 (0.885 – 1.045), 0.361	1.042 (0.948 – 1.145), 0.393
Sex	1.230 (0.757 – 2.000), 0.403	1.468 (0.769 – 2.801), 0.244	0.987 (0.381 – 2.558), 0.979	2.210 (0.788 – 6.193), 0.132
Education	1.002 (0.922 - 1.088), 0.967	1.006 (0.905 – 1.119), 0.905	0.935 (0.807 – 1.084), 0.373	1.123 (0.928 – 1.360), 0.232
MEM	0.976 (0.699 – 1.364), 0.888	0.727 (0.353 – 1.495), 0.386	0.741 (0.284 – 1.933), 0.540	0.892 (0.254 – 3.134), 0.858
Aß42 (pg/ml)	1.000 (0.999 – 1.001), 0.748	1.000 (0.999 – 1.001), 0.969	1.001 (1.000 – 1.003), 0.072	0.999 (0.997 – 1.001), 0.261
t-tau (pg/ml)	0.999 (0.996 – 1.001), 0.256	0.999 (0.996 – 1.003), 0.594	1.001 (0.996 – 1.006), 0.622	0.997 (0.990 – 1.003), 0.259
p-tau (pg/ml)	1.015 (0.995 – 1.035), 0.140	1.003 (0.974 – 1.033), 0.833	0.986 (0.948 – 1.026), 0.495	1.024 (0.971 – 1.079), 0.386

Note. *Aβ42* beta-amyloid 42; *CO* healthy controls; *CI* Confidence Interval; *MEM* Memory factor score; *OR* Odds ratio; *p-tau* phospho-tau; *REL* first degree relatives of AD patients; *SCD* subjective cognitive decline; *t-tau* total tau

Table S2. B) Regression Coefficients and Odds Ratios (95% Confidence Intervals) from binary logistic regression to predict a GAI-SF score ≥ 2

	Whole sample (n = 302)	Cognitively healthy (CO+SCD+REL) (n = 194)	Cognitively healthy worrier (n = 104)	Cognitively healthy non-worrier (n = 88)
Predictors	**OR (95% CI), p-value**	**OR (95% CI), p-value**	**OR (95% CI), p-value**	**OR (95% CI), p-value**
Age	0.973 (0.927 – 1.021), 0.264	0.958 (0.897 – 1.023), 0.200	0.941 (0.866 – 1.022), 0.147	0.934 (0.783 – 1.115), 0.452
Sex	1.513 (0.872 – 2.625), 0.141	1.730 (0.820 – 3.650), 0.150	1.112 (0.431 – 2.870), 0.827	6.204 (0.772 – 49.876), 0.086
Education	1.014 (0.923 – 1.115), 0.772	1.029 (0.909 – 1.165), 0.650	0.992 (0.854 – 1.153), 0.921	1.095 (0.806 – 1.488), 0.560
MEM	1.162 (0.794 – 1.701), 0.439	0.625 (0.283 – 1.379), 0.245	0.695 (0.279 – 1.727), 0.433	0.504 (0.045 – 5.621), 0.578
Aß42 (pg/ml)	1.000 (0.999 – 1.001), 0.569	1.000 (0.999 – 1.001), 0.836	1.002 (1.000 – 1.003), 0.022*	0.996 (0.993 – 1.000), 0.060
t-tau (pg/ml)	1.000 (0.997 – 1.002), 0.899	0.999 (0.995 – 1.004), 0.796	1.001 (0.996– 1.006), 0.707	1.000 (0.989 – 1.011), 0.979
p-tau (pg/ml)	1.011 (0.990 – 1.032), 0.316	1.001 (0.967 – 1.037), 0.940	0.996 (0.957 – 1.036), 0.832	0.984 (0.892 – 1.085), 0.747

Note. * $p < .05$. *Aβ42* beta-amyloid 42; *CO* healthy controls; *CI* Confidence Interval; *MEM* Memory factor score; *OR* Odds ratio; *p-tau* phospho-tau; *REL* first degree relatives of AD patients; *SCD* subjective cognitive decline; *t-tau* total tau

Table S3. Regression Coefficients and Odds Ratios (95% Confidence Intervals) from binary logistic regression to predict the presence of agitation, depression, anxiety, apathy, irritability and sleep disturbances on the NPI-Q.

		Whole sample (n = 296)	Cognitively healthy (CO+SCD+REL) (n = 190)	Cognitively healthy worrier (n = 100)	Cognitively healthy non-worrier (n = 88)
	Predictors	**OR (95% CI), p-value**	**OR (95% CI), p-value**	**OR (95% CI), p-value**	**OR (95% CI), p-value**
**Agitation**	Age	1.047 (0.991 – 1.106), 0.102	1.105 (1.011 – 1.208), 0.028*	1.153 (1.022 – 1.301), 0.021*	1.114 (0.917 – 1.353), 0.278
	Sex	0.445 (0.232 – 0.856), 0.015**	0.346 (0.113 – 1.057), 0.063	0.361 (0.092 – 1.422), 0.145	0.122 (0.010 – 1.520), 0.102
	Education	0.968 (0.871 – 1.077), 0.554	0.987 (0.838 – 1.162), 0.876	1.021 (0.839 – 1.242), 0.836	0.885 (0.610 – 1.285), 0.521
	MEM	1.054 (0.702 – 1.582), 0.800	0.723(0.256 – 2.042), 0.541	1.266 (0.337 – 4.750), 0.727	0.133 (0.007 – 2.400), 0.172
	Aß42 (pg/ml)	0.998 (0.997 – 1.000), 0.009**	0.999 (0.997 – 1.000), 0.085	0.997 (0.995 – 1.000), 0.020*	1.002 (0.998 – 1.006), 0.275
	t-tau (pg/ml)	1.000 (0.997 – 1.003), 0.995	0.999 (0.993 – 1.004), 0.632	0.996 (0.989 – 1.002), 0.198	1.009 (0.996 – 1.023), 0.167
	p-tau (pg/ml)	1.010 (0.988 – 1.033), 0.372	1.010 (0.966 – 1.055), 0.674	1.042(0.990 – 1.096), 0.112	0.881 (0.767 – 1.012), 0.073
**Depression**	Age	1.013 (0.958 – 1.070), 0.656	1.021 (0.933 – 1.116), 0.654	0.973 (0.874 – 1.084), 0.623	
	Sex	1.332 (0.700 – 2.533), 0.382	0.805(0.287 – 2.256), 0.680	0.974 (0.296 – 3.207), 0.966	
	Education	1.019 (0.914 – 1.135), 0.739	0.965 (0.816 – 1.141), 0.675	0.989 (0.818 – 1.195), 0.905	
	MEM	0.718 (0.477 – 1.080), 0.111	0.762 (0.282 – 2.057), 0.592	0.951(0.326 – 2.777), 0.927	**< 5 cases**
	Aß42 (pg/ml)	0.998 (0.997 – 0.999), 0.004**	0.998 (0.996 – 1.000), 0.044*	0.998 (0.996 – 1.000), 0.132	
	t-tau (pg/ml)	0.999 (0.997 – 1.002), 0.700	1.001 (0.995 – 1.006), 0.849	1.002 (0.996 – 1.008), 0.582	
	p-tau (pg/ml)	1.003 (0.979 – 1.028), 0.785	0.986 (0.938 – 1.036), 0.568	0.981 (0.930 – 1.035), 0.488	
**Anxiety**	Age	0.995 (0.935 – 1.060), 0.885	1.082 (0.972 – 1.205), 0.151	1.075 (0.937 – 1.234), 0.304	
	Sex	0.763 (0.372 – 1.562), 0.459	0.741 (0.210 – 2.613), 0.641	0.584 (0.122 – 2.785), 0.500	
	Education	0.891 (0.782 – 1.015), 0.082	0.857 (0.694 – 1.060), 0.155	0.831 (0.640 – 1.078), 0.164	
	MEM	0.762 (0.483 – 1.201), 0.242	0.900 (0.290 – 2.790), 0.855	1.023 (0.298 – 3.507), 0.971	**< 5 cases**
	Aß42 (pg/ml)	0.999 (0.997 – 1.000), 0.046*	0.997 (0.995 – 1.000), 0.027*	0.996 (0.992 – 0.999), 0.022*	
	t-tau (pg/ml)	0.999 (0.995 – 1.002), 0.391	0.996 (0.989 – 1.003), 0.265	0.995 (0.986 – 1.003), 0.197	
	p-tau (pg/ml)	1.011 (0.984 – 1.039), 0.421	1.032 (0.977 – 1.090), 0.264	1.041 (0.977 – 1.108), 0.216	

Table S3 (Cont.).

	Predictors	Whole sample (n = 296) OR (95% CI), p-value	Cognitively healthy (CO+SCD+REL) (n = 190) OR (95% CI), p-value	Cognitively healthy worrier (n = 100) OR (95% CI), p-value	Cognitively healthy non-worrier (n = 88) OR (95% CI), p-value
**Apathy**	Age	1.014 (0.948 – 1.085), 0.680	1.199 (1.013 – 1.420), 0.035*	1.253 (0.987 – 1.592), 0.064	**< 5 cases**
	Sex	0.358 (0.156 – 0.823), 0.016*	0.088 (0.008 – 0.967), 0.047*	0.104 (0.007 – 1.658), 0.109	
	Education	0.896 (0.781 – 1.029), 0.119	0.764 (0.565 – 1.032), 0.080	0.848 (0.621 – 1.158), 0.299	
	MEM	0.566 (0.348 – 0.922), 0.022*	1.226 (0.162 – 9.256), 0.844	3.771 (0.21 – 65.507), 0.362	
	Aß42 (pg/ml)	0.999 (0.998 – 1.001), 0.203	0.998 (0.996 – 1.001), 0.219	0.996 (0.991 – 1.001), 0.133	
	t-tau (pg/ml)	1.000 (0.997 – 1.003), 0.994	0.996 (0.987 – 1.005), 0.399	0.998 (0.989 – 1.006), 0.617	
	p-tau (pg/ml)	1.005 (0.978 – 1.032), 0.739	1.046 (0.981 – 1.116), 0.171	1.051 (0.983 – 1.124), 0.147	
**Irritability**	Age	1.072 (1.016 – 1.130), 0.011*	1.111 (1.029 – 1.119), 0.007	1.170 (1.051 – 1.303), 0.004**	1.034 (0.880 – 1.216), 0.680
	Sex	0.384 (0.204 – 0.722), 0.003**	0.455 (0.181 – 1.147), 0.095	0.407 (0.122 – 1.362), 0.145	0.340 (0.060 – 1.945), 0.225
	Education	0.953 (0.859 – 1.056), 0.358	1.014 (0.879 – 1.169), 0.849	0.983 (0.825 – 1.171), 0.848	0.967 (0.705 – 1.328), 0.837
	MEM	1.154 (0.766 – 1.738), 0.494	0.739 (0.300 – 1.817), 0.510	1.183 (0,371 – 3.777), 0.776	0.317 (0.041 – 2.470), 0.273
	Aß42 (pg/ml)	0.999 (0.998 – 1.000), 0.073	0.999 (0.997 – 1.000), 0.098	0.998 (0.996 – 1.000), 0.042*	1.002 (0.998 – 1.006), 0.345
	t-tau (pg/ml)	0.997 (0.994 – 1.000), 0.051	0.995 (0.989 – 1.000), 0.048*	0.996 (0.989 . 1.002), 0.150	0.988 (0.976 – 1.002), 0.085
	p-tau (pg/ml)	1.031 (1.007 – 1.055), 0.020*	1.038 (0.995 – 1.082), 0.081	1.039 (0.992 – 1.088), 0.107	1.020 (0.910 – 1.143), 0.736
**Sleep disturbances**	Age	0.989 (0.937 – 1.043), 0.679	1.016 (0.946 – 1.090), 0.663	0.982 (0.894 – 1.078), 0.699	1.055 (0.923 – 1.205), 0.431
	Sex	2.006 (1.091 – 3.690), 0.025*	3.403 (1.418 – 8.166), 0.006**	3.451 (1.120 – 10.630), 0.031*	5.683 (1.203 – 26.842), 0.028*
	Education	0.975 (0.878 – 1.083), 0.635	1.010 (0.884 – 1.155), 0.879	0.998 (0.844 – 1.180), 0.982	1.037 (0.800 – 1.343), 0.768
	MEM	0.886 (0.584 – 1.344), 0.570	0.515 (0.210 – 1.258), 0.145	0.842 (0.303 – 2.344), 0.742	0.135 (0.021 – 0.864), 0.034*
	Aß42 (pg/ml)	1.000 (0.999 – 1.001), 0.523	1.000 (0.998 – 1.001), 0.585	0.999 (0.997 – 1.001), 0.236	1.002 (0.999 – 1.004), 0.183
	t-tau (pg/ml)	0.998 (0.995 – 1.001), 0.294	0.998 (0.993 – 1.003), 0.383	0.997 (0.991 – 1.004), 0.429	1.000 (0.992 – 1.008), 0.946
	p-tau (pg/ml)	1.005 (0.979 – 1.031), 0.724	1.018 (0.980 – 1.056), 0.357	1.022 (0.975 – 1.070), 0.367	0.994 (0.924 – 1.070), 0.880

Note. * $p < .05$, ** $p < .01$. *Aβ42* beta-amyloid 42; *CO* healthy controls; *CI* Confidence Interval; *MEM* Memory factor score; *OR* Odds ratio; *p-tau* phospho-tau; *REL* first degree relatives of AD patients; *SCD* subjective cognitive decline; *t-tau* total tau
